# Genomic analyses of microdissected Hodgkin and Reed-Sternberg cells: mutations in epigenetic regulators and p53 are frequent in refractory classic Hodgkin lymphoma

**DOI:** 10.1038/s41408-019-0195-7

**Published:** 2019-03-11

**Authors:** Elena Mata, Sara Fernández, Aurora Astudillo, Rubén Fernández, Mónica García-Cosío, Margarita Sánchez-Beato, Mariano Provencio, Mónica Estévez, Carlos Montalbán, Miguel A. Piris, Juan F. García

**Affiliations:** 1 Departments of Pathology, MD Anderson Cancer Center Madrid, Madrid, Spain; 2 Departments of Pathology, Hospital Universitario de Cabueñes, Gijón, Spain; 3 Departments of Hematology, Hospital Universitario de Cabueñes, Gijón, Spain; 4 Department of Pathology, Hospital Universitario Ramón y Cajal, Madrid, Spain; 5 Instituto Investigación Sanitaria Puerta de Hierro – Segovia de Arana (IDIPHISA), Centro de Investigación Biomédica en Red de Cáncer (CIBERONC) Instituto de Salud Carlos III, Madrid, Spain; 6 Departments of Hematology, MD Anderson Cancer Center Madrid, Madrid, Spain; 7 Department of Pathology, Fundación Jiménez Díaz, Madrid; Centro de Investigación Biomédica en Red de Cáncer (CIBERONC) Instituto de Salud Carlos III, Madrid, Spain

Classical studies have consistently identified some specific gene mutations in classic Hodgkin lymphoma (cHL), mainly affecting members of the NF-kappaB and JAK/STAT pathways^1–4^. However, this knowledge is yet to be exploited in the clinical milieu. Molecular analyses of cHL have been limited because of the paucity of Hodgkin and Reed-Sternberg (HRS) cells, which usually account for < 5% of cells as identified by standard CD30 immunostaining. Various enrichment strategies, such as laser capture microdissection (LCM), could facilitate the identification of genetic alterations^5^. Also, in recent years, the sensitivity and specificity of next-generation sequencing (NGS) techniques have been greatly improved by simultaneously testing selected genes, arranged in comprehensive gene panels. This is even possible with formalin-fixed, paraffin-embedded (FFPE) tissue samples.

However, very few NGS studies have described the genomic landscape of the disease. The first whole-exome sequencing analyses of primary Hodgkin and Reed-Sternberg (HRS) cells found beta-2-microglobulin (*B2M*) to be the most commonly altered gene^6^. Recently, our group have identified concurrent genetic lesions in relevant signaling pathways, such as those of JAK-STAT, NF-kappaB, and BCR, as well as in epigenetic regulators^7^. These findings are largely concordant with other NGS studies in cHL cell lines^8^. More recently, Tiacci et al^9^ have reported frequent mutations affecting genes of the JAK-STAT pathway, as well as mutations in *GNA13*, *XPO1*, and *ITPKB*. However, none of these works set out to identify variants associated with response to treatment.

The identification of specific genetic lesions and better biological characterization of the subgroup of patients with refractory disease remain major research goals. Here we analyze the genomic characteristics of 12 cHL tumors, corresponding to selected patients with diseases that are primary refractory to conventional therapy, and compare the genetic variants identified in the HRS cells from primary tumors and relapsed tumors.

Initially, formalin-fixed paraffin-embedded (FFPE) tumor samples and clinical data from 20 cHL patients were obtained from the records of the participating institutions. All patients were intentionally selected because of their refractoriness to conventional treatment: primary-progressive disease (absence of complete remission after treatment) or early relapse (less than 12 months after complete remission). All cases received ABVD therapy. We collected representative tumor blocks from both, the original pretreatment and the relapse biopsy. All the samples and data were collected through the MDACC Madrid Biobank, in accordance with the technical and ethical procedures of the Spanish National Biobank Network, including anonymization processes and obtaining written informed consent according to the Helsinki Declaration. Approval was obtained from the institutional review board (Clinical Research Ethical Committee, ref. 354/12). Clinical characteristics of the patients are summarized in Table 1. After LCM, DNA extraction and library construction, 12 cases fulfilled the minimum quality criteria and the generated libraries were sequenced and their data analyzed.

**Table 1:** Clinical characteristics of the cases

Case #	Histological subtype	Bone marrow involvement	Bulky mass	Ann Arbor stage	Age	Sex	IPS	First-line therapy	Response	Follow-up
1	MC	no	yes (mediastinal)	IIB	23	F	0	ABVD (x4) + Rt	CR + R	DOD (13 months)
2	NS	no	yes (mediastinal)	IIB	31	F	4	ABVD (x6) + Rt	CR + R	Alive (90 months)
3	MC	no	yes (retroperitoneal)	IIIB	27	M	1	ABVD (x6)	CR + R	Alive (64 months)
4	NS	NA	no	IIA	40	M	2	ABVD (x4)	CR + R	Alive (84 months)
5	NS	no	no	IIB	41	M	2	ABVD (x4)	PD	DOD (36 months)
6	NS	no	yes	IIB	32	F	3	ABVD (x6) + Rt	PD	DOD (10 months)
7	NS	no	no	IIB	59	M	4	ABVD (x6)	PD	DOD (7 months)
8	NS	yes	yes (mediastinal)	IV	23	F	2	ABVD (x6) + Rt	PD	AWD (42 months)
9	NS	no	no	IIIA	42	M	2	ABVD (x6)	PD	DOD (27 months)
10	MC	yes	no	IV	21	M	5	ABVD (x6)	PD	Lost
11	MC	yes	yes (abdominal)	IV	69	F	3	ABVD (x6) + Rt	CR + R	AWD (32 months)
12	NS	yes	no	IV	73	M	4	ABVD (x6)	PD	DOD (15 months)

*MC* mixed cellularity, *NS* nodular sclerosis, *IPS* International Prognostic Score, *ABVD* adriamycin, bleomycin, vinblastine, and dacarbazine, *Rt* radiotherapy, *CR* + *R* complete remission and early relapse, *PD* progressive disease, *DOD* dead of disease, *AWD* alive with disease

We enriched the tumor cells by LCM using a Palm MicroBeam V4 microscope (Carl Zeiss Inc., Oberkochen, Germany) equipped with a catapult system for contamination-free sample isolation. In brief, 5-μm adjacent sections from FFPE tissues were cut, deparaffinized and immunostained with anti-CD30 antibody (clone BerH2, Dako/Agilent, Madrid, Spain). Parallel sections were also stained with hematoxylin and eosin to assess morphology, content and distribution of HRS cells.

All experimental procedures were repeated in the original pretreatment biopsy and the relapse biopsy, comparing each duplicated sample and discarding non-concordant variants. From each case, 15,000–25,000 individually picked HRS cells duplicated per case were isolated from FFPE tissue. We also isolated normal lymphocytes from the reactive background (morphologically normal lymphocytes, CD30-negative, duplicated per case). This procedure was repeated in the original pretreatment biopsy and the relapse biopsy. Thus, eight different experiments were analyzed by NGS for each patient (all the workflow is depicted in Supplementary Figure 1).

After cell enrichment, we performed targeted analysis of 35 genes involved in B cell-related pathways (Supplemental Table 1), previously reported as being the most frequently mutated in cHL^6,7,9^, using Ion Torrent PGM (Thermo Fisher Scientific, NY, USA) technology with a modified protocol from that previously published^7^. DNA was extracted from duplicates of isolated HRS cells and CD30-negative fractions using the Gentra Puregene Tissue Kit (Qiagen, Germantown, MD). Libraries were constructed starting with 10 ng of genomic DNA and following the manufacturer´s protocol. Sequencing (BAM) files have been deposited in the NCBI Sequence Repository (SRA: PRJNA506444). The data were analyzed with the Torrent Suite program. All variants were examined with Integrative Genomics Viewer (IGV) software^10^, discarding non-concordant variants. Functional consequences of the SNVs were predicted using the publicly available PROVEAN (shift and polyphen-2) and CONDEL algorithms.

Using this protocol for targeted sequencing, and after filtering SNPs, non-concordant, and silent variants, 42 candidate somatic SNVs were identified in the CD30-positive fraction and 15 in the CD30-negative fraction, from the 238 initial candidate variants (Fig. 1 and Supplementary Table 2 summarize the results after comparing each duplicated sample and discarding non-concordant variants). These figures could be related to genetic instability^11^, extensive clonal diversity and mutations with very low variant allele frequencies, as described in HRS cells^7^ and tumors^9^, or could arise because some of the individual variants are sequencing errors. An astringent filtering process, and the fact that, in all cases, the analysis was done in duplicate, guarantee that the risk of false-positive results is very low, even at the cost of losing valuable information about low-frequency mutations and intratumor heterogeneity.

**Fig. 1:**
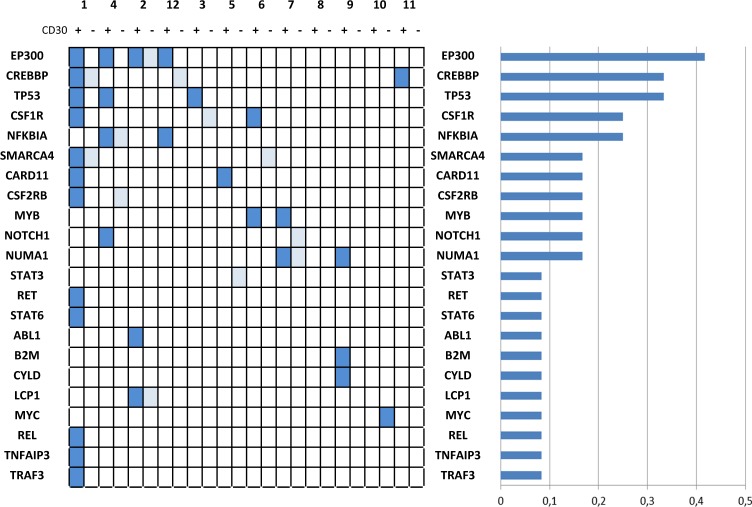
Distribution and frequency of variants. NGS analyses were repeated in the initial pretreatment and the relapse biopsies, comparing duplicated samples and discarding non-concordant variants. Light blue indicates variants detected in the CD30-negative fraction. The histogram on the right indicates relative frequencies

Most cases presented gene variants that had previously been described in cHL^6,7,9^. We confirmed the previously reported prevalent mutations affecting the NF-kappaB pathway, associated with JAK/STAT activation, *B2M* mutations, and mutations affecting the BCR pathway.

Interestingly, mutations affecting the *TP53* gene were overrepresented in this series of refractory cases (3 out of 12 cases, 25%), with some variants that are redundantly detected in different cases, such as C238Y (cases 1 and 3) or Y234* (cases 3 and 4). These results contrast with classic studies that focused solely on p53 pathway mutations in cHL, and previous NGS studies of unselected cHL only identified rare *TP53* mutations (Supplementary Table 3). It is worth noting that *TP53* mutations have been more frequently described recently in refractory cHL through the use of more sensitive NGS techniques^12^. This is entirely unsurprising since mutations of the p53 pathway are a classic prognostic factor associated with chemoresistance in most cancers.

In addition, the most frequently mutated genes, *EP300* and *CREBBP*, correspond to epigenetic regulators. Identical variants were detected in different cases and also in the CD30-positive and CD30-negative fractions, such as *EP300* N1776H or *CREBBP* P1083L. *EP300* encodes a transcriptional coactivator protein and functions as a histone acetyltransferase and regulates transcription via chromatin remodeling. Confirming previous results^7,9^, mutations in the histone acetylation domains of *EP300* and *CREBBP* are frequent in cHL (one or both alterations are present in 5 out of 12 cases, 41.6%), similar to what is seen in DLBCL and follicular lymphoma. Furthermore, a recent report has presented evidence that histone acetyltransferase might act as a tumor suppressor that controls MHCII expression and promote tumor immune control and evasion^13^, and that drugs targeting histone acetyltransferase activities and chromatin remodeling, such as BET inhibitors, represent a novel therapeutic approach that have recently demonstrated efficacy in solid malignancies and lymphomas.

This study is obvious limited by the small number of cases, experimental complexity, and the impossibility of analyzing a parallel cohort of samples of “good responders” as control patients. Nevertheless, it seems clear that mutational frequencies are different from the distribution reported in previous unselected series (Supplementary Table 3). However, in a very recent study using a targeted NGS panel commercially available in archival tumor samples particularly enriched with relapsed patients, the most commonly mutated gene was also *TP53*, detected in 11 patients (22%). Possible comparisons are limited by the obvious methodological differences between the various studies.

In our previous work^7^, in which 34% of the cases were primary refractory cHL, *EP300* was also one of the most frequently mutated genes (occurring in 14% of cases). There were no statistically differences between mutational patterns of responders and non-responders in the original analyses, probably due to the small sample size. Nevertheless, further Kaplan–Meier analyses of failure-free survival (FFS), using the records from the 46 patients for whom we have complete clinical information and follow-up, revealed a significant association between the presence of *CREBBP* mutations and FFS (Supplemental Figure 2). Although we must recognize that a formal comparison between these different studies has obvious limitations.

An important observation in our study is the discovery of several mutations in the CD30-negative fraction in most of the experiments, using an LCM protocol with a very low probability of being subject to common contamination. We can speculate that these results might indicate a clonally related population, in a stem cell or less differentiated state, that is not morphologically or phenotypically distinguishable, as other authors have previously suggested^14^. In any case, additional experiments are necessary to corroborate this finding and fully understand its pathogenic significance.

In conclusion, it seems that several recurrent mutational events are present in primary refractory cHL that could be used as biomarkers and eventually exploited for therapy. *TP53* mutations seem to represent a relevant predictive marker also in cHL, and the highly prevalent mutations of epigenetic regulators *EP300* and *CREBBP* suggest a rationale for alternative therapeutic strategies that need to be investigated further.

## Supplementary information

Supplementary Appendix
