# Supplementary material for: Genomic analyses of microdissected Hodgkin and Reed-Sternberg cells: mutations in epigenetic regulators and p53 are frequent in refractory classic Hodgkin lymphoma
Source: Blood Cancer J. 2019 Mar 11;9(3):34. doi: 10.1038/s41408-019-0195-7 (PMC6411728; doi:10.1038/s41408-019-0195-7)
Supplement: Supplementary file 1 — Supplementary Appendix [file 41408_2019_195_MOESM1_ESM.docx]

Genomic analyses of microdissected Hodgkin and Reed-Sternberg cells: mutations in epigenetic regulators and p53 are frequent in refractory classic Hodgkin lymphoma

Elena Mata (1), Sara Fernández (1), Aurora Astudillo (3); Rubén Fernández (4), Mónica García-Cosío (5), Margarita Sánchez-Beato (6), Mariano Provencio (6), Mónica Estévez (2), Carlos Montalbán (2), Miguel A. Piris (7), and Juan F. García (1).

Supplementary Information

**Supplementary Table 1. Ampliseq panel.** For NGS, libraries were constructed using an Ampliseq Custom Panel, including the 35 selected genes distributed in 1214 amplicons. Sample emulsion PCRs and enrichment were performed using the PGM Hi-Q OT2 Kit, and the libraries were sequenced using 318 v2 chips with Ion PGM Sequencing 200 v2 (Thermo Fisher Scientific).

| **Gene name** | **Chromosome** | **Number of amplicons** | **Total bases** | **Covered bases** | **Missed bases** | **Overall coverage** | **Number of exons** |
| --- | --- | --- | --- | --- | --- | --- | --- |
| **ABL1** | chr9 | 44 | 3649 | 3444 | 205 | 0.944 | 12 |
| **B2M** | chr15 | 6 | 390 | 390 | 0 | 1.000 | 3 |
| **BCL10** | chr1 | 9 | 732 | 621 | 111 | 0.848 | 3 |
| **BTK** | chrX | 29 | 2272 | 2272 | 0 | 1.000 | 21 |
| **CARD11** | chr7 | 53 | 3705 | 3418 | 287 | 0.923 | 24 |
| **CASP8** | chr2 | 24 | 1813 | 1801 | 12 | 0.993 | 12 |
| **CD19** | chr16 | 26 | 1814 | 1760 | 54 | 0.970 | 15 |
| **CD38** | chr4 | 18 | 983 | 983 | 0 | 1.000 | 8 |
| **CREBBP** | chr16 | 91 | 7639 | 6496 | 1143 | 0.850 | 31 |
| **CSF1R** | chr5 | 47 | 3129 | 2877 | 252 | 0.919 | 21 |
| **CSF2** | chr5 | 7 | 475 | 433 | 42 | 0.912 | 4 |
| **CSF2RB** | chr22 | 36 | 2824 | 2486 | 338 | 0.880 | 13 |
| **CYLD** | chr16 | 41 | 3041 | 2953 | 88 | 0.971 | 18 |
| **EP300** | chr22 | 90 | 7555 | 7143 | 412 | 0.945 | 31 |
| **FAS** | chr10 | 17 | 1098 | 1085 | 13 | 0.988 | 10 |
| **IL32** | chr16 | 10 | 627 | 572 | 55 | 0.912 | 8 |
| **LCP1** | chr13 | 30 | 2034 | 2034 | 0 | 1.000 | 15 |
| **MYB** | chr6 | 34 | 2446 | 2413 | 33 | 0.987 | 18 |
| **MYC** | chr8 | 15 | 1395 | 1318 | 77 | 0.945 | 3 |
| **NFKBIA** | chr14 | 12 | 1014 | 841 | 173 | 0.829 | 6 |
| **NFKBIE** | chr6 | 17 | 1563 | 1227 | 336 | 0.785 | 6 |

**Supplementary Table 1. Ampliseq panel (2).**

| **Gene name** | **Chromosome** | **Number of amplicons** | **Total bases** | **Covered bases** | **Missed bases** | **Overall coverage** | **Number of exons** |
| --- | --- | --- | --- | --- | --- | --- | --- |
| **NOTCH1** | chr9 | 95 | 8008 | 6527 | 1481 | 0.815 | 34 |
| **NUMA1** | chr11 | 78 | 6598 | 6179 | 419 | 0.936 | 25 |
| **PIK3CD** | chr1 | 43 | 3355 | 2950 | 405 | 0.879 | 22 |
| **PTPN1** | chr20 | 21 | 1408 | 1403 | 5 | 0.996 | 11 |
| **REL** | chr2 | 25 | 1970 | 1733 | 237 | 0.880 | 11 |
| **RET** | chr10 | 47 | 3577 | 3162 | 415 | 0.884 | 21 |
| **SH3BP5** | chr3 | 19 | 1458 | 1402 | 56 | 0.962 | 10 |
| **SMARCA4** | chr19 | 73 | 5399 | 5048 | 351 | 0.935 | 37 |
| **SOCS1** | chr16 | 6 | 646 | 520 | 126 | 0.805 | 1 |
| **STAT3** | chr17 | 37 | 2543 | 2492 | 51 | 0.980 | 25 |
| **STAT6** | chr12 | 42 | 2754 | 2653 | 101 | 0.963 | 22 |
| **TNFAIP3** | chr6 | 29 | 2453 | 2367 | 86 | 0.965 | 8 |
| **TP53** | chr17 | 22 | 1383 | 1351 | 32 | 0.977 | 15 |
| **TRAF3** | chr14 | 21 | 1807 | 1704 | 103 | 0.943 | 10 |

**Supplementary Table 2. Summary of single nucleotide variants concordant in the initial and relapse samples.**

Summary of variants after filtering; variants identified in the CD30-negative fraction are highlighted in gray. The data were analyzed with the Torrent Suite program using the “variant caller” plug-in program, selecting the somatic low-stringency mode to detect single nucleotide variants (SNVs) with low allele frequency. We restricted the analyses to variants with a total coverage of at least 100x (average coverage 452.7, range 109-1972). Additionally, and to eliminate erroneous base calling, all variants were examined with Integrative Genomics Viewer (IGV) software, comparing each duplicated sample and discarding non-concordant variants. Sequencing (BAM) files have been deposited in the NCBI Sequence Repository (SRA: SUB4823073).

| **Sample** | **Fraction** | **Gene ID** | **Chrom** | **Position** | **Change** | **Frequency** | **Original coverage** | **Type** | **Aminoacid change** |
| --- | --- | --- | --- | --- | --- | --- | --- | --- | --- |
| HL1 | CD30(+) | CARD11 | chr7 | 2968317 | G>A | 3,2 | 772 | SNP | R557C |
| HL1 | CD30(+) | CARD11 | chr7 | 2978383 | C>T | 12,8 | 133 | SNP | R316K |
| HL1 | CD30(+) | CARD11 | chr7 | 2983941 | C>T | 7,3 | 165 | SNP | V197M |
| HL1 | CD30(+) | CARD11 | chr7 | 2985542 | A>G | 11,7 | 206 | SNP | V90A |
| HL1 | CD30(+) | CREBBP | chr16 | 3781305 | G>A | 11 | 162 | SNP | S1717F |
| HL1 | CD30(+) | CREBBP | chr16 | 3817813 | G>A | 40,8 | 222 | SNP | P1083L |
| HL1 | CD30(+) | CSF1R | chr5 | 149440443 | C>T | 4 | 693 | SNP | G651R |
| HL1 | CD30(+) | CSF1R | chr5 | 149450132 | T>C | 38,1 | 428 | SNP | H362R |
| HL1 | CD30(+) | CSF2RB | chr22 | 37334416 | C>T | 9,8 | 502 | SNP | Q862* |
| HL1 | CD30(+) | EP300 | chr22 | 41573041 | A>C | 5,8 | 311 | SNP | N1776H |
| HL1 | CD30(+) | REL | chr2 | 61147736 | G>A | 5,6 | 266 | SNP | G349D |
| HL1 | CD30(+) | RET | chr10 | 43619120 | T>C | 3,4 | 855 | SNP | W935R |
| HL1 | CD30(+) | RET | chr10 | 43622048 | C>T | 10,3 | 465 | SNP | T1022I |
| HL1 | CD30(+) | SMARCA4 | chr19 | 11134254 | C>A | 13,3 | 266 | SNP | P1038T |
| HL1 | CD30(+) | STAT6 | chr12 | 57492671 | G>A | 8,9 | 189 | SNP | P657L |
| HL1 | CD30(+) | STAT6 | chr12 | 57496258 | C>T | 10,2 | 147 | SNP | A443T |
| HL1 | CD30(+) | TNFAIP3 | chr6 | 138192497 | C>T | 11,6 | 129 | SNP | R45* |
| HL1 | CD30(+) | TP53 | chr17 | 7577568 | C>T | 31,9 | 367 | SNP | C238Y |
| HL1 | CD30(+) | TRAF3 | chr14 | 103372023 | T>G | 6,4 | 204 | SNP | F537V |
| HL1 | CD30(-) | CREBBP | chr16 | 3817813 | G>A | 40,8 | 222 | SNP | P1083L |
| HL1 | CD30(-) | SMARCA4 | chr19 | 11134254 | C>A | 10 | 1240 | SNP | P1038T |
| HL2 | CD30(+) | ABL1 | chr9 | 133730409 | G>A | 10,1 | 109 | SNP | G178S |
| HL2 | CD30(+) | EP300 | chr22 | 41547886 | C>T | 12,9 | 171 | SNP | S956F |
| HL2 | CD30(+) | EP300 | chr22 | 41573041 | A>C | 3 | 885 | SNP | N1776H |
| HL2 | CD30(+) | EP300 | chr22 | 41573936 | T>C | 11,2 | 160 | SNP | L2074P |
| HL2 | CD30(-) | EP300 | chr22 | 41572413 | C>T | 8 | 627 | SNP | Q1648* |
| HL2 | CD30(-) | EP300 | chr22 | 41573041 | A>C | 7,7 | 298 | SNP | N1776H |

**Supplementary Table 2. Summary of single nucleotide variants concordant in the initial and relapse samples. (2)**

| **Sample** | **Fraction** | **Gene ID** | **Chrom** | **Position** | **Change** | **Frequency** | **Original coverage** | **Type** | **Aminoacid change** |
| --- | --- | --- | --- | --- | --- | --- | --- | --- | --- |
| HL2 | CD30(-) | LCP1 | chr13 | 46721070 | G>A | 5,2 | 384 | SNP | Q383* |
| HL3 | CD30(+) | TP53 | chr17 | 7577568 | C>T | 18,7 | 1064 | SNP | C238Y |
| HL3 | CD30(+) | TP53 | chr17 | 7577579 | G>C | 8,5 | 528 | SNP | Y234* |
| HL3 | CD30(-) | CSF1R | chr5 | 149440491 | T>C | 9,3 | 279 | SNP | K635E |
| HL4 | CD30(+) | EP300 | chr22 | 41574302 | G>A | 7,9 | 126 | SNP | G2196E |
| HL4 | CD30(+) | NFKBIA | chr14 | 35871233 | G>A | 8,1 | 262 | SNP | R314C |
| HL4 | CD30(+) | NOTCH1 | chr9 | 139413157 | C>T | 4,5 | 823 | SNP | G329S |
| HL4 | CD30(+) | TP53 | chr17 | 7577579 | G>C | 12,6 | 247 | SNP | Y234* |
| HL4 | CD30(+) | TP53 | chr17 | 7578268 | A>C | 6,4 | 140 | SNP | L194R |
| HL4 | CD30(-) | CSF2RB | chr22 | 37325765 | G>A | 10,7 | 122 | SNP | V212I |
| HL4 | CD30(-) | NFKBIA | chr14 | 35872541 | G>A | 3,7 | 215 | SNP | T121I |
| HL5 | CD30(+) | CARD11 | chr7 | 2977642 | C>T | 11 | 136 | SNP | E348K |
| HL5 | CD30(+) | CARD11 | chr7 | 2978351 | G>A | 12,2 | 123 | SNP | Q327* |
| HL5 | CD30(-) | STAT3 | chr17 | 40474420 | C>A | 35 | 256 | SNP | D661Y |
| HL5 | CD30(-) | STAT3 | chr17 | 40476800 | C>T | 9,9 | 161 | SNP | W510* |
| HL6 | CD30(+) | CSF1R | chr5 | 149447806 | AGC>- | 5 | 1768 | DEL | L531. |
| HL6 | CD30(+) | MYB | chr6 | 135518445 | C>T | 13,6 | 301 | SNP | P517L |
| HL6 | CD30(-) | SMARCA4 | chr19 | 11134254 | C>A | 8 | 281 | SNP | P1038T |
| HL7 | CD30(+) | MYB | chr6 | 135524425 | C>T | 5,4 | 295 | SNP | T711I |
| HL7 | CD30(+) | NUMA1 | chr11 | 71717267 | A>G | 31,9 | 382 | SNP | Y1836H |
| HL7 | CD30(-) | NOTCH1 | chr9 | 139396319 | T>C | 13 | 339 | SNP | H1840R |
| HL7 | CD30(-) | NUMA1 | chr11 | 71717267 | A>G | 31,9 | 382 | SNP | Y1836H |
| HL9 | CD30(+) | B2M | chr15 | 45003808 | C>T | 12,4 | 1030 | SNP | Q22* |
| HL9 | CD30(+) | CYLD | chr16 | 50788275 | C>T | 18,2 | 482 | SNP | L285F |
| HL9 | CD30(+) | NUMA1 | chr11 | 71727231 | C>T | 27,3 | 882 | SNP | E440K |
| HL10 | CD30(-) | MYC | chr8 | 128750605 | CAG>- | 6,6 | 420 | DEL | Q48. |
| HL11 | CD30(+) | CREBBP | chr16 | 3781294 | T>C | 7,5 | 1050 | SNP | M1721V |
| HL12 | CD30(+) | EP300 | chr22 | 41553257 | C>T | 18,5 | 627 | SNP | Q1116* |
| HL12 | CD30(+) | NFKBIA | chr14 | 35872550 | G>A | 14,8 | 1792 | SNP | A118V |
| HL12 | CD30(-) | CREBBP | chr16 | 3781292 | C>T | 5,1 | 415 | SNP | M1721I |

**Supplementary Table 3. Main results of previous studies using NGS in cHL tumor samples.** In previous unselected series the clinical outcome of the patients was not described,^6,9^ and mutations of the chromatin-modifying genes CREBBP and EP300 or TP53 were only occasionally observed.

| **Series** | **N** | **Sequencing techniques** | **Enrichment** | **Clinical features** | **Results** |
| --- | --- | --- | --- | --- | --- |
| Reichel et al^6^ | 10 | WES | Flow sorting | N.A., unselected samples | *B2M* most frequently mutated  *CREBBP* mutated in 1 case (10%)  No *EP300* or *TP53* mutations |
| Tiacci et al^9^ | 34 | WES | LCM | N.A., unselected samples | *STAT6* most frequently mutated  *TP53* mutated in 3 cases (9%)  Very rare *CREBBP* mutations (1 case)  No *EP300* mutations |
| Mata et al^7^ | 57 | Targeted NGS panel | Selection of HRS-rich areas | Unselected samples  34% refractory cHL | *EP300, BTK, and STAT6* most frequently mutated *EP300* mutated in 12%; associated with FFS  Very rare *CREBBP* mutations (1 case)  No *TP53* mutations |
| Liang et al^12^ | 49 | Targeted NGS panel | None | Mostly refractory cHL  Relapsed samples | TP53 most frequently mutated (22%)  No *CREBBP* or *EP300* mutations |

WES: whole-exome sequencing

**Supplementary Figure 1.** Analysis pipeline and overall results.


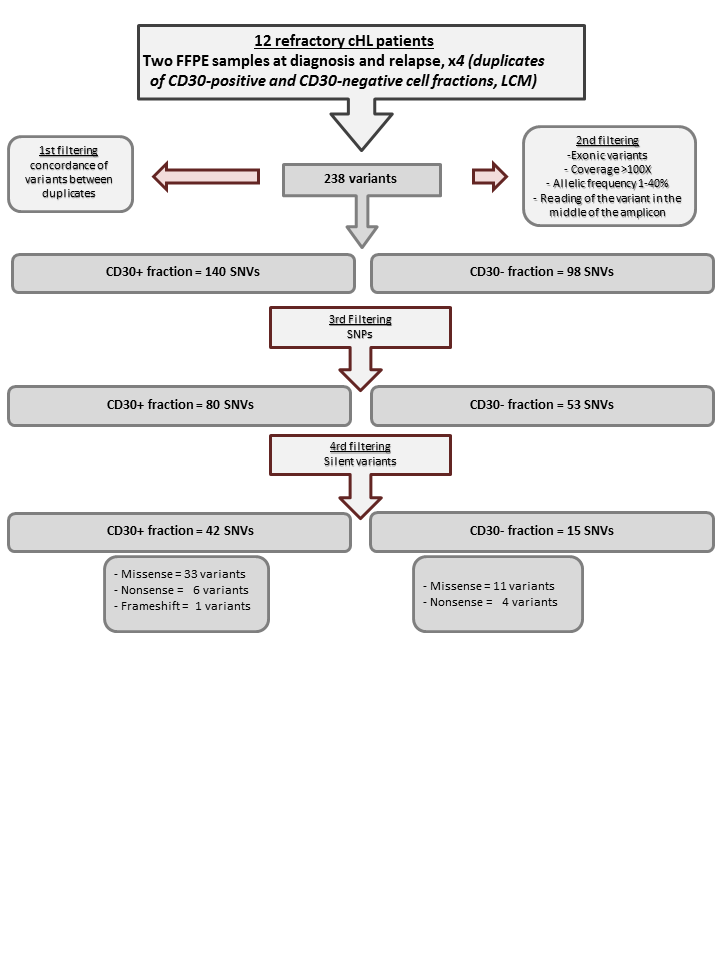


**Supplementary Figure 2.**

Clinical information and follow-up was available for 46 of the 57 cases included in our previous report.^7^ Correlations between the presence of SNVs in *TP53*, *EP300*, and *CREBBP* genes and failure-free survival (FFS) were estimated by the Kaplan–Meier method and the curves were compared using the log-rank test. Differences were considered statistically significant for values of P<0.05. Statistical analyses were performed using SPSS version 17.0 (SPSS Inc., Chicago, IL).
